# Therapeutic endocannabinoid augmentation for mood and anxiety disorders: comparative profiling of FAAH, MAGL and dual inhibitors

**DOI:** 10.1038/s41398-018-0141-7

**Published:** 2018-04-26

**Authors:** Gaurav Bedse, Rebecca J. Bluett, Toni A. Patrick, Nicole K. Romness, Andrew D. Gaulden, Philip J. Kingsley, Niels Plath, Lawrence J. Marnett, Sachin Patel

**Affiliations:** 1 Department of Psychiatry and Behavioral Sciences, Vanderbilt University Medical Center, Nashville, TN USA; 2 Vanderbilt Brain Institute, Vanderbilt University, Nashville, TN USA; 3 Departments of Biochemistry, Chemistry, and Pharmacology, A.B. Hancock Jr. Memorial Laboratory for Cancer Research, Vanderbilt Institute of Chemical Biology, Vanderbilt University School of Medicine, Nashville, TN USA; 4 H. Lundbeck A/S, Copenhagen, Denmark; 5 Department of Molecular Physiology and Biophysics, Vanderbilt University School of Medicine, Nashville, TN USA

## Abstract

Recent studies have demonstrated anxiolytic potential of pharmacological endocannabinoid (eCB) augmentation approaches in a variety of preclinical models. Pharmacological inhibition of endocannabinoid-degrading enzymes, such as fatty acid amide hydrolase (FAAH) and monoacylglycerol lipase (MAGL), elicit promising anxiolytic effects in rodent models with limited adverse behavioral effects, however, the efficacy of dual FAAH/MAGL inhibition has not been investigated. In the present study, we compared the effects of FAAH (PF-3845), MAGL (JZL184) and dual FAAH/MAGL (JZL195) inhibitors on (1) anxiety-like behaviors under non-stressed and stressed conditions, (2) locomotor activity and body temperature, (3) lipid levels in the brain and (4) cognitive functions. Behavioral analysis showed that PF-3845 or JZL184, but not JZL195, was able to prevent restraint stress-induced anxiety in the light–dark box assay when administered before stress exposure. Moreover, JZL195 treatment was not able to reverse foot shock-induced anxiety-like behavior in the elevated zero maze or light–dark box. JZL195, but not PF-3845 or JZL184, decreased body temperature and increased anxiety-like behavior in the open-field test. Overall, JZL195 did not show anxiolytic efficacy and the effects of JZL184 were more robust than that of PF-3845 in the models examined. These results showed that increasing either endogenous AEA or 2-AG separately produces anti-anxiety effects under stressful conditions but the same effects are not obtained from simultaneously increasing both AEA and 2-AG.

## Introduction

Mood and anxiety disorders are chronic, disabling conditions that impose enormous cost both on individuals and society^1^. Current clinical treatments for anxiety and mood disorders are primarily based on augmenting monoaminergic transmission^2^. Current treatment approaches are often only partially effective and are often associated with adverse effects^3^. The search for novel pharmacological treatments for these conditions is driven by the growing need for improved efficacy, tolerability and side effect profiles. Over the past 10 years, molecular, cellular, physiological and pharmacological studies have moved the field of anxiety and stress-related disorder research beyond the monoamine hypothesis^4,5^. 

The endocannabinoid (eCB) system has gained attention in recent years as a potential target for novel anxiolytics^6,7^. The eCB system is a retrograde lipid signaling system that is implicated in the regulation of multiple physiological functions in the nervous system^8^. A number of preclinical studies support the role of the eCB system as a modulator of anxiety-related behaviors, depressive-like behaviors and extinction of fear memories^9–11^. Anandamide (*N*-arachidonylethanolamine [AEA]) and 2-arachidonoylglycerol (2-AG) are two major eCBs that exert biological effects via activation of type 1 and 2 cannabinoid receptors (CB1R and CB2R)^12,13^. The psychoactive component of Cannabis sativa, Δ^9^-tetrahydrocannabinol (Δ^9^-THC) and other CB1 receptor agonists have been studied for their effects on anxiety-like behaviors. It has been shown that at low doses Δ^9^-THC and CB1 receptor agonists exert anxiolytic effects in various preclinical models of anxiety-like phenotypes^14–17^. However, direct CB1 agonists can also produce a range of side effects such as motor impairments, catalepsy, hypothermia and cognitive impairments^18,19^. Therefore, an alternate approach to avoid the adverse effects of direct CB1 agonist has been to focus on eCB modulation.

AEA and 2-AG are degraded by fatty acid amide hydrolase (FAAH) and monoacylglycerol lipase (MAGL) enzymes, respectively^20^. Selective FAAH (PF-3845) and MAGL (JZL184) inhibitors have been developed which elevate AEA and 2-AG levels in the brain, respectively^21,22^. Along with others, we have previously shown that the pharmacological inhibition of eCB-degrading enzymes elicit promising anxiolytic effects in a variety of preclinical anxiety models without serious adverse behavioral effects^9,23,24^. Recently, we showed that a pharmacological and functional redundancy between AEA and 2-AG signaling exist in the modulation of anxiety-like behaviors^25^. However, the full spectrum of cannabimimetic activities is not observed upon inhibition of either FAAH or MAGL alone, but dual FAAH/MAGL inhibition produces effects more similar to direct CB1 agonists. The discovery of the dual FAAH/MAGL inhibitor JZL195 has provided the possibility of exploring the anxiolytic effects of dual FAAH/MAGL inhibition ^26^.

To our knowledge, there are no comprehensive studies examining the comparative effects of dual FAAH/MAGL inhibition with selective FAAH or MAGL inhibition on anxiety-like behaviors. Thus, in this study we explored the comparative effects of FAAH, MAGL and dual FAAH/MAGL inhibitors on anxiety-like behaviors, locomotor activity, body temperature and cognitive functions. This study aimed to gain a clearer understanding of the effect of concomitant increases in AEA and 2-AG levels on anxiety-like behaviors.

## Materials and methods

### Animals

All studies were carried out in accordance with the National Institute of Health Guide for the Care and Use of Laboratory Animals, and approved by the Vanderbilt University Institutional Animal Care and Use Committee. All mice were group housed on a 12:12 light–dark cycle (lights on at 6:00 a.m.) with food and water available ad libitum. All behavioral testing was performed between 6:00 a.m. and 6:00 p.m. Male ICR (CD-1) mice 6–9 weeks of age were used for all experiments (Envigo, Indianapolis, IN, USA) and female ICR mice 6 weeks old were only used for foot shock experiment. Male C57BL/6J mice 8–9 weeks old were used only for elevated zero maze (EZM) experiment. Male and female mice were single housed for at least 1 week prior to behavioral testing for the foot shock experiments and group housed for the rest of the studies.

### Drugs and treatment

The drugs used were FAAH inhibitor PF-3845 (0.1, 1 and 10 mg kg^–^^1^), MAGL inhibitor JZL184 (5, 8, 10 and 40 mg kg^–1^) and FAAH/MAGL dual inhibitor JZL195 (5, 10 and 40 mg kg^–1^)^25^. All drugs were administered by intraperitoneal injection at a volume of 1 ml kg^–1^ in the vehicle dimethyl sulfoxide. Drugs were administered 2 h prior to behavioral testing. The doses, pretreatment time and route of administration were chosen on the basis of our previous studies ^25,27^.

### Stress exposure

#### Restraint stress

Mice were brought into the behavioral room daily and subjected to tube restraint for 30 min in modified transparent 50-ml plastic conical tubes with numerous small air holes to increase ventilation (between 9:00 a.m. and 1:00 p.m.)^28^. Mice entered the tubes head first and air holes were concentrated toward the conical end. A plug was inserted and secured snugly behind the mouse to restrict movement. Control mice were left undisturbed in their home cages, except for tail marking at the beginning of the experiment and as needed to maintain identifying marks throughout the protocol. Mice were tested for anxiety-like behavior using the light–dark exploration test immediately after restraint stress exposure.

#### Foot shock stress

Foot shock stress occurred 24 h before behavioral testing and consisted of six 0.7 mA foot-shocks delivered 1 min apart using a MED Associates fear-conditioning chamber (St. Albans, VT, USA). Each 2-s shock coincided with the last 2 s of a 30-s auditory tone. Twenty-four hours after foot shock stress, mice were tested in EZM and light–dark box.

### Light–dark box test

The light–dark test was performed as previously described^25^. Mice were individually placed into sound-attenuating chambers (27.9 × 27.9 cm; MED-OFA-510; MED Associates, St. Albans, VT, USA) containing dark box inserts that split the chamber into light (250–400 lux) and dark ( < 5 lux) halves (Med Associates ENV-511). Beam breaks from 16 infrared beams were recorded by Activity Monitor v5.10 (MED Associates) to monitor position and behavior during the 10-min testing period.

### Novelty-induced hypophagia

The novelty-induced hypophagia (NIH) test consisted of 4 training days in the home cage and 1 test day in a novel cage. Home cages and bedding were not changed for the duration of the experiment. Group housed mice were habituated to testing rooms illuminated by red light (< 50 lux) for at least 30 min. During training days, mice were given access to a highly palatable substance (liquid vanilla Ensure, Abbott Laboratories, Abbott Park, IL, USA) in their home cages for 30 min. On novel cage testing day, mice were habituated in red light for 60 min and then each mouse was transferred to a new, empty cage in a brightly lit room (~300 lux) with 30 min access to liquid vanilla Ensure during which latency to drink and total consumption were recorded.

### Elevated zero maze test

The EZM (San Diego instruments, California, USA) is an annular white platform divided into four equal quadrants. It consists of two open arms and two closed arms enclosed by tall external walls. The outer and inner diameters of the EZM are 60.9 cm and 50.8 cm, respectively. The apparatus was elevated 60.9 cm from the floor. Light levels in the open arms were approximately 200 lux, whereas the closed arms were < 100 lux. Mice were placed in the middle of an open arm of the maze, and allowed to explore for 5 min. ANY-maze (Stoelting, Wood Dale, Illinois, USA) video-tracking software was used to monitor and analyze behavior during the test.

### Open-field

For open-field testing (OFT), exploration of a novel open-field arena contained within a sound-attenuating chamber was monitored for 30 min (27.9 × 27.9 × 20.3 cm; MED-OFA-510; MED Associates, St. Albans, Vermont). The walls of the open-field arena were made of clear plexiglass; this arena was contained within an opaque sound-attenuating chamber. Beam breaks from 16 infrared beams were recorded by Activity Monitor v5.10 (MED Associates) to monitor position and behavior.

### Morris water maze test

The Morris water maze (MWM) test was performed as previously described^29^ with some small modifications. Mice were trained for 5 consecutive days before the probe trial. Each day of training consisted of four trials with an intertrial interval of 30 min. Mice were placed in the maze at semi-randomized start points each day such that one trial each day was from each of the four start locations (N, S, E and W). Mice were allowed to remain on the platform for 10-s before being removed from the maze. If the mouse did not reach the escape platform within 60 s it was gently guided to the escape platform. Between each trial, mice were dried in cages with paper towels and heating pads and then returned to their home cages. On the first day of training, the platform was indicated by a flag protruding from the water to acclimate the mice to the apparatus and climbing onto the platform. On the day of the probe trial, the escape platform was removed and mice were allowed to swim in the pool for 60 s. Animal movements and location was recorded using Anymaze.

### Barnes-maze test

The Barnes-maze is a white 90 cm diameter circular plastic platform containing 12 holes (5 cm diameter) evenly spaced around the perimeter. Mice were placed in a floorless start box in the middle of the maze for a 10-s acclimation period. The start box was then lifted to release the mouse and initiate the test. The target hole led to an escape box where mice were allowed to sit for at least 15-s before being returned to their home cage. If a mouse did not find the escape hole during the 3-min trial it was gently guided there. Mice underwent four trials per day for a 4-day training period with a 10- to 15-min intertrial interval. On the fifth day, mice underwent a 60-s probe trial during which the escape hole was blocked. Animal movements and location were recorded using Anymaze.

### Statistics

Statistical significance was calculated using a two-tailed unpaired *t*-test, one-way or two-way analysis of variance (ANOVA) with post hoc Holm–Sidak’s multiple comparisons test as noted in figure legends. All statistical analyses were conducted using Prism Graphpad 6 (San Diego, CA, USA). For behavioral studies, all replicates (*n* values) represent biological replicates defined as data derived from a single mouse and *n* values are mentioned in the figures. Data are presented as mean ± SEM. unless otherwise stated in the figure legends. *P* < 0.05 was considered significant throughout. *F* and *P*-values for ANOVA are indicated within figure panels, whereas post hoc significance level is indicated above individual bars or time points. *R*^2^ and *P*-values for linear regression analyses are shown in all correlation panels. Rout test for outlier identification was used. Testing was counterbalanced, but no randomization was performed, and sample sizes were derived empirically during the course of the experiments guided by our previous work using these assays. Experimenters were blinded to treatment condition during experimentation.

### Lipid analysis

Lipid analysis was performed as described previously^25^. The samples were analyzed for AEA, 2-AG, arachidonic acid (AA), *N*-oleoylethanolamine (OEA) and their deuterated internal standards on an liquid chromatography and mass spectrometry (LC-MS) system consisting of a Shimadzu Nexera UPLC system in-line with a SCIEX 6500 QTrap mass spectrometer. The tissue collection and parameters for the chromatographic regime are as described previously ^25^.

## Results

### FAAH and MAGL inhibition, but not dual FAAH/MAGL inhibition, prevents restraint stress-induced anxiety-like behavior in the light–dark box

**Fig. 1:**
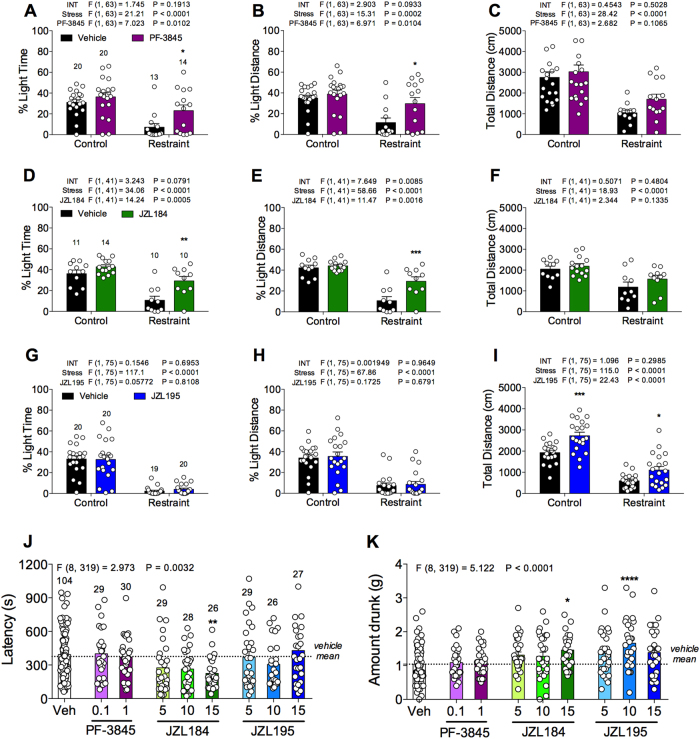
Comparative effects of PF-3845, JZL184 and JZL195 on restraint stress-induced anxiety-like behavior in the light–dark box or novelty-induced anxiety-like behavior in the NIH assay. The effects of **a**-**c** selective FAAH inhibitor PF-3845 (1 mg kg^–1^), **d**-**f** selective MAGL inhibitor JZL184 (10 mg kg^–1^) and **g**-**i** dual FAAH/MAGL inhibitor JZL195 (10 mg kg^–1^) systemic administration on the percent light time, percent light distance and total distance traveled in 10 min in the light–dark box assay. The effects of PF-3845 (0.1 and 1 mg kg^–1^), JZL184 (5, 10 and 15 mg kg^–1^) and JZL195 (5, 10 and 15 mg kg^–1^) on the novel cage **j** latencies and **k** consumptions in NIH assay. Significant *F* and* P*-values from one-way and two-way analysis of variance noted above bar graphs; *P < 0.05, **P < 0.01, ***P < 0.001, vs. respective vehicle-treated group by Holm–Sidak post hoc multiple comparisons test in bar graphs. Data are presented as means ± SEM. NIH novelty-induced hypophagia

To examine the effects of FAAH, MAGL or dual FAAH/MAGL inhibitors in the regulation of anxiety, we tested the FAAH inhibitor PF-3845 (1 mg kg^–1^), MAGL inhibitor JZL184 (10 mg kg^–1^) or dual FAAH/MAGL inhibitor JZL195 (10 mg kg^–1^) in the light–dark box assay. We have previously validated the light–dark box assay under both basal and stressed condition by using diazepam, a standard anxiolytic drug^25^. Examination of the population distribution of light time revealed a normal distribution (KS normality test, *P* > 0.1000). Under basal conditions, none of the compounds affected the percent light time or percent light distance (Figs. 1a-i). However, JZL195, but not PF-3845 or JZL184, significantly increased total distance traveled (Fig. 1i).

Thirty minutes of restraint stress significantly reduced percent light time, percent light distance and total distance traveled compared with control mice in the light–dark box assay (Figs. 1a-i). Systemic administration of PF-3845 or JZL184, but not JZL195, 90 min before stress exposure reduced these stress-induced changes in behavior compared with vehicle-treated stressed mice. The stress-induced reduction of percent light time and percent light distance were prevented by PF-3845 (Figs. 1a, b) or JZL184 treatment (Figs. 1d, e) but not by JZL195 (Figs. 1g, h). Moreover, JZL195 significantly increased total distance traveled in stressed mice (Fig. 1i).

### MAGL inhibition, but not FAAH or dual FAAH/MAGL inhibition, prevents novelty-induced anxiety-like behavior in the NIH assay

Next, we examined the effects of PF-3845 (0.1 and 1 mg kg^–1^), JZL184 (5, 10, 15 mg kg^–1^) or JZL195 (5, 10, 15 mg kg^–1^) on novelty-induced anxiety-like behaviors by using the NIH assay, which is highly sensitive to stress and eCB manipulations^30^. The highest dose of JZL184 significantly reduced latency to consume palatable food in the novel cage test, compared with vehicle-treated mice (Fig. 1j). JZL184 also increased consumption of palatable food (Fig. 1k). JZL195 did not decrease latency to consume palatable food (Fig. 1j). However, JZL195 (10 mg kg^–1^) significantly increased consumption of palatable food (Fig. 1k). PF-3845 neither decreased latency nor increased consumption of the palatable food.

### Dual FAAH/MAGL inhibition does not reverse foot shock-induced anxiety-like behavior in the EZM test or light–dark box

**Fig. 2:**
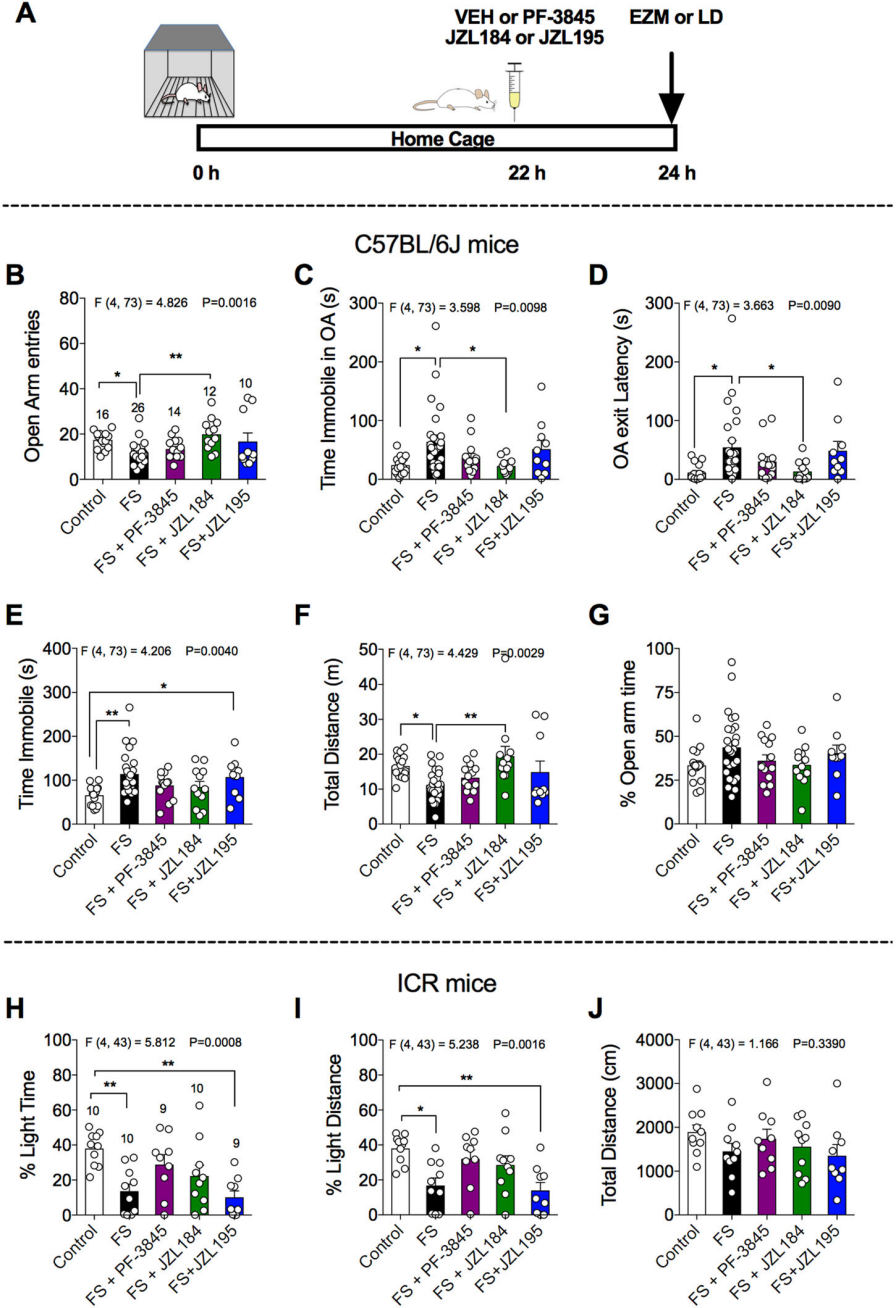
Comparative effects of PF-3845, JZL184 and JZL195 on foot shock-induced anxiety-like behavior in the elevated zero maze or light–dark box. **a** Schematic diagram depicts the timeline of the experiment. The effects of PF-3845 (1 mg kg^–1^), JZL184 (10 mg kg^–1^) and JZL195 (10 mg kg^–1^) systemic administration on the **b** open arm entries, **c** time immobile in open arm, **d** open arm exit latency, **e** total time immobile, **f** total distance and **g** % open arm time in the EZM. The effects of PF-3845 (1 mg kg^–1^), JZL184 (10 mg kg^–1^) and JZL195 (10 mg kg^–1^) systemic administration on the **h** percent light time, **i** percent light distance and **j** total distance traveled in 10 min in the light–dark box assay. Significant *F* and *P-*values from one-way analysis of variance noted above bar graphs; *P < 0.05, **P < 0.01, vs. stress group by Holm–Sidak post hoc multiple comparisons test in bar graphs. Data are presented as means ± SEM. EZM elevated zero maze, LD light–dark box, FS foot shock

Thus far, our data suggest that MAGL inhibition decreases the latency to consume palatable food and increases consumption of palatable food in the novel cage test under non-stressed conditions and both MAGL and FAAH inhibition are able to prevent restraint-induced reductions in the percent light time and light distance when administered before stress exposure. Next, we wanted to examine whether these inhibitors could reverse the effects of stress on anxiety-like behavior if administered after stress exposure. To examine this, we exposed C57BL/6j or ICR mice to foot shock stress 24 h before behavioral testing using EZM or light–dark box, respectively. Mice were injected with PF-3845 (1 mg kg^–1^), JZL184 (10 mg kg^–1^) or JZL195 (10 mg kg^–1^) 22 h after stress exposure as shown in Fig. 2a and 2 h later, subjected to either EZM or light–dark box assay. The effects of foot shock stress on the various parameters of EZM test are shown in Figs. 2b-g. Behavioral analysis revealed a significant effect of foot shock exposure on open arm entries (Fig. 2b), time immobile in open arm (Fig. 2c), open arm exit latency (Fig. 2d), time immobile (Fig. 2e) and total distance traveled (Fig. 2f). Further, post hoc analyses revealed that JZL184 treatment significantly increased open arm entries (Fig. 2b) and total distance traveled (Fig. 2f), and decreased the total time immobile in the open arms (Fig. 2c) and open arm exit latency (Fig. 2d) compared with vehicle-treated foot shock stressed mice. However, PF-3845 and JZL195 were not able to reverse foot shock-induced anxiety-like behavior.

The effects of foot shock stress on the various parameters of the light–dark box test in ICR males are shown in Figs. 2h-j. Foot shock exposure significantly reduced percent light time and light distance compared with the control male mice. Systemic administration of PF-3845 or JZL184, but not JZL195, ameliorated stress-induced decrease in percent light time and light distance. The percent light time and light distance of stressed, PF-3845 or JZL184-treated mice were not significantly different from the control mice, but this reversal of anxiety-like behaviors was incomplete, as these groups were also not significantly different from the stressed, vehicle-treated mice. However, the percent light time and light distance of stressed, JZL195-treated mice were significantly decreased compared with control mice. We also performed similar experiment on the ICR female mice, however, the variability was more in foot shock exposed female mice of the tested cohort (Fig. S1). The sex-specific effects of PF-3845, JZL184 and JZL195 need to study in detail in future.

### Dual FAAH/MAGL, but not FAAH or MAGL, inhibition decreases body temperature and increases anxiety-like behavior in the OFT

Next, to examine potential adverse effects of PF-3845, JZL184 or JZL195 on locomotor activity and body temperature (two well-established effects of direct cannabinoid agonists), we tested the effects of the lowest therapeutic dose and 10-fold higher dose of PF-3845, or the maximum soluble dose in the case of JZL184 and JZL195. Two hours after systemic administration, mice were tested in the OFT followed immediately by body temperature measurement via rectal probe. Mice were then sacrificed and brains were collected for lipid analysis.

**Fig. 3:**
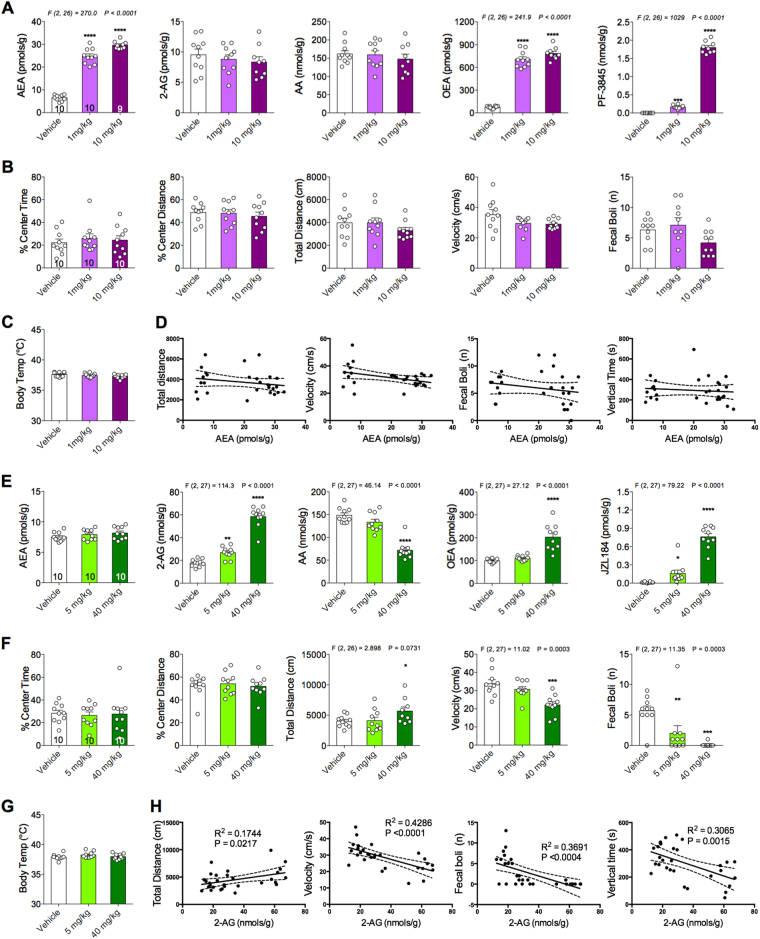
Effects of PF-3845 and JZL184 on brain endocannabinoid levels, locomotor activity, anxiety-like behavior and body temperature. **a** Acute fatty acid amide hydrolase inhibition (PF-3845) effects on brain *N*-arachidonylethanolamine (AEA), 2-arachidonoylglycerol (2-AG), arachidonic acid (AA) and oleoylethanolamine (OEA) levels. **b** Effects of PF-3845 on % center time, % center distance, total distance, average velocity and number of fecal boli in the open-field test. **c** PF-3845 treatment did not affect body temperature. **d** Brain AEA correlations with various behavioral parameters. **e** Acute monoacylglycerol lipase inhibition (JZL184) effects on brain AEA, 2-AG, arachidonic acid (AA) and oleoylethanolamine (OEA) levels. **f** Effects of JZL184 on % center time, % center distance, total distance, average velocity and number of fecal boli in the open-field test. **g** JZL184 treatment did not affect body temperature. **h** Brain 2-AG correlations with various behavioral parameters. Significant *F* and *P-*values from one-way analysis of variance noted above bar graphs; *P < 0.05, **P < 0.01, ***P < 0.001, ****P < 0.0001 vs vehicle group by Holm–Sidak post hoc multiple comparisons test in bar graphs. Linear regression (solid line) with 95% confidence intervals (dashed lines) shown in figures. Data are presented as means ± SEM

In line with previous studies^22,27^, PF-3845 increased brain AEA and OEA without altering brain 2-AG and AA (Fig. 3a). PF-3845 was detected at high levels in brain after i.p. injection (Fig. 3a). PF-3845 (1 and 10 mg kg^–1^) did not affect the percent center time, percent center distance, total distance, average velocity or number of fecal boli (Fig. 3b). Moreover, neither dose of PF-3845 altered body temperature (Fig. 3c). Brain AEA levels were not correlated with total distance traveled, velocity, fecal boli or vertical time (Fig. 3d).

Systemic administration of JZL184 (5 and 40 mg kg^–1^) significantly increased brain 2-AG levels dose-dependently (Fig. 3e). In line with previous studies^25,30^, elevations in brain 2-AG levels were accompanied by significant reductions in the levels of AA (Fig. 3e). Brain AEA levels were unaffected by JZL184 but OEA brain levels were significantly increased by the highest dose of JZL184. JZL184 was detected at high levels in the brain after i.p. injection (Fig. 3e). Moreover, the highest dose of JZL184 increased total distance traveled and decreased average velocity compared with vehicle-treated mice (Fig. 3f). JZL184 administration significantly reduced the number of fecal boli dose-dependently (Fig. 3f). JZL184 did not change body temperature at either dose tested (Fig. 3g). Brain 2-AG levels are positively correlated with total distance traveled and negatively correlated with velocity, number of fecal boli and vertical time (Fig. 3h).

**Fig. 4:**
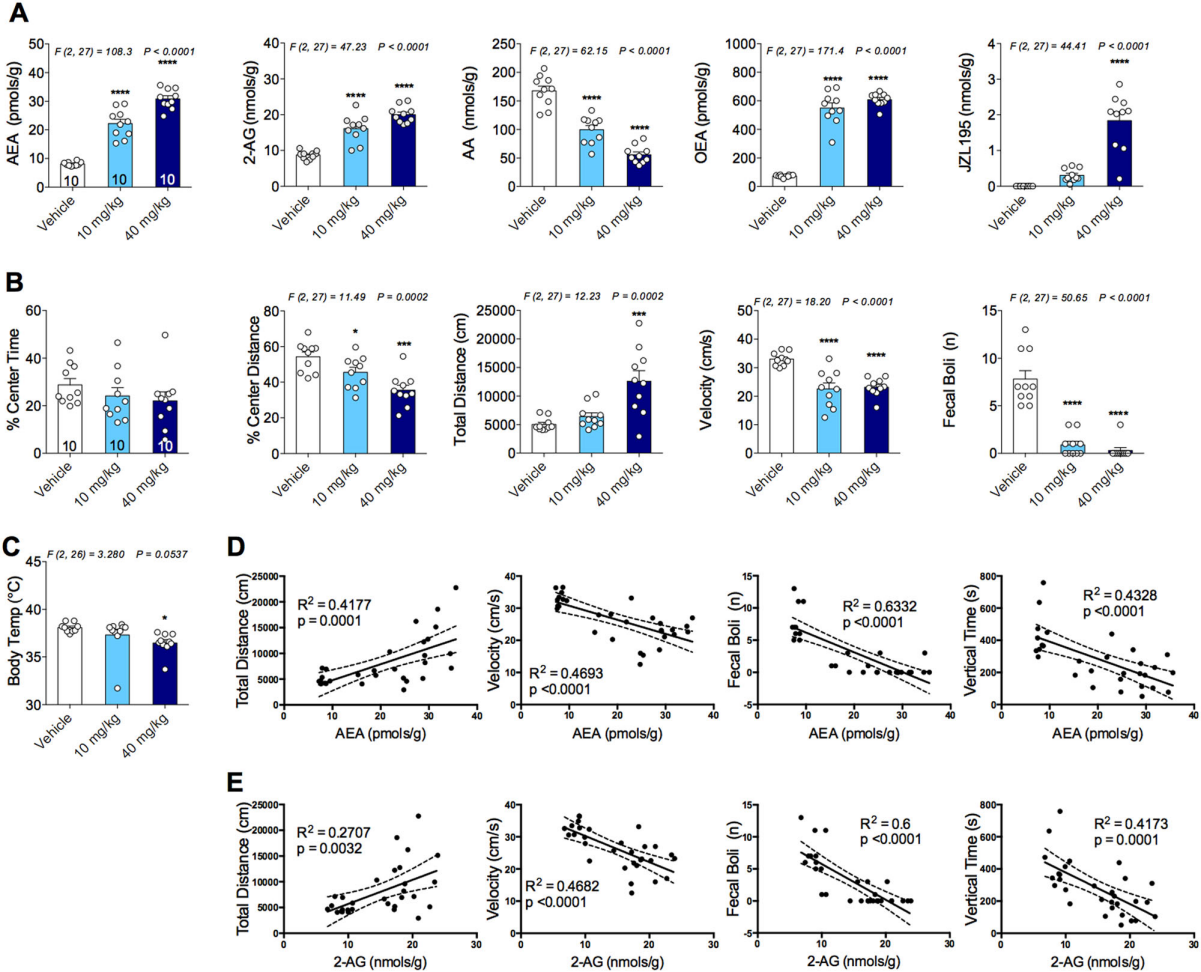
Effects of JZL195 on brain endocannabinoid levels, locomotor activity, anxiety-like behavior in the open-field test and body temperature. **a** Dual fatty acid amide hydrolase and monoacylglycerol lipase inhibition effects on brain *N*-arachidonylethanolamine (AEA), 2-arachidonoylglycerol (2-AG), arachidonic acid (AA) and oleoylethanolamine (OEA) levels. **b** Effects of JZL195 on % center time, % center distance, total distance, average velocity and number of fecal boli in the open-field test. **c** JZL195 treatment dose-dependently lowered body temperature. **d** Brain AEA and **e** 2-AG correlations with various behavioral parameters. Significant *F* and *P*-values from one-way analysis of variance noted above bar graphs; *P < 0.05, ***P < 0.001, ****P < 0.0001 vs vehicle group by Holm–Sidak post hoc multiple comparisons test in bar graphs. Linear regression (solid line) with 95% confidence intervals (dashed lines) shown in figures. Data are presented as means ± SEM

Systemic administration of JZL195 (10 and 40 mg kg^–1^) significantly increased both brain AEA and 2-AG levels (Fig. 4a). As expected, JZL195 also decreased AA and increased OEA levels in the brain (Fig. 4a). JZL195 was detected at high levels in the brain after i.p. injection (Fig. 4a). JZL195 significantly decreased percent center distance, velocity and number of fecal boli (Fig. 4b). Mice treated with the highest dose of JZL195 traveled a significantly greater distance compared with vehicle-treated mice (Fig. 4b). Moreover, JZL195 also decreased body temperature dose dependently (Fig. 4c). Both brain AEA and 2-AG levels are positively correlated with distance traveled and negatively correlated with velocity, number of fecal boli and vertical time (Figs. 4d, e).

### FAAH, MAGL and dual FAAH/MAGL inhibition do not impair cognitive function in the MWM or Barnes-maze test

**Fig. 5:**
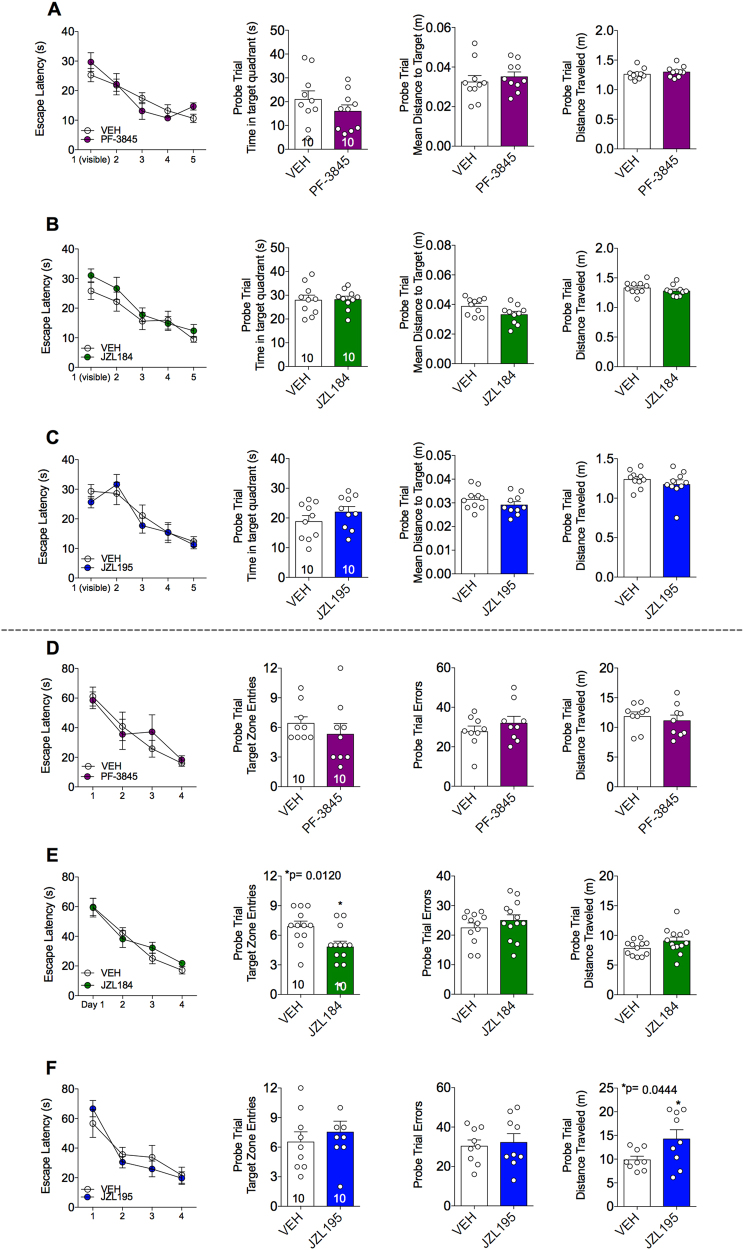
Comparative effects of PF-3845, JZL184 and JZL195 on Morris water maze or Barnes-maze performance. The effects of **a** PF-3845 (1 mg kg^–1^), **b** JZL184 (8 mg kg^–1^) and **c** JZL195 (10 mg kg^–1^) on escape latency during training days, time spent in target quadrant, mean distance to target and total distance traveled in Morris water maze test. The effects of **d** PF-3845 (1 mg kg^–1^), **e** JZL184 (8 mg kg^–1^) and **f** JZL195 (10 mg kg^–1^) on escape latency during training days, target zone entries, probe trial errors and total distance traveled in the Barnes-maze test. Significant *P-*values from *t*-test noted above bar graphs; *P < 0.05, vs vehicle group by unpaired *t-*test in bar graphs. Data are presented as means ± SEM

It is well established that learning and memory can be impaired by cannabinoids^31,32^. In order to establish whether acute modulation of eCB levels also impairs cognitive function, we used the MWM and Barnes-maze to evaluate the effects of PF-3845, JZL184 or JZL195 on learning and memory. Escape latency decreases significantly across training trials, demonstrating that mice learned both tasks well (Figs. 5a-f). Systemic administration of PF-3845 (1 mg kg^–1^), JZL184 (8 mg kg^–1^) or JZL195 (10 mg kg^–1^), administered before the probe trials, did not alter the escape latency in either test. None of the compounds showed any effect on time spent in the target quadrant, mean distance to target or distance traveled in the MWM test (Figs. 5a-c). Also, none of the treatments showed any effect on target zone entries or probe trial errors in the Barnes-maze test except JZL184, which reduced target zone entries (Figs. 5d-f). However, the probe trial errors (number of times mice explored incorrect target holes) and distance traveled were not different in the JZL184-treated mice compared with the vehicle-treated mice (Fig. 5e). Only JZL195 treatment increased the path length to the escape box, but other parameters such as zone entries and probe trial errors were not different from vehicle-treated mice (Fig. 5f). As mice were not treated during acquisition, and only during probe trial testing (which is a measure of memory recall), it remains possible that acute eCB manipulations could affect spatial learning. Additionally, as others have suggested, it is also possible that chronic treatment with these inhibitors could impact cognitive function. Clearly more testing is needed to resolve these conflicting reports, but our data confirm that acute, indirect eCB enhancement does not affect memory recall at doses that may be relevant for treating anxiety-like dysfunction.

## Discussion

The main findings of this study are that (1) restraint stress-induced anxiety-like behavior can be prevented by either acute selective FAAH or MAGL, but not by dual FAAH/MAGL, inhibition, (2) acute MAGL inhibition decreases novelty-induced anxiety and can reverse foot shock-induced anxiety-like behavior, (3) dual FAAH/MAGL, but not FAAH or MAGL, inhibition decreases body temperature and increases anxiety-like behavior and (4) none of the inhibitors impaired cognitive functions at doses relevant for anxiety-like behavior.

In this study, we found that FAAH, MAGL and dual FAAH/MAGL inhibition had little or no effect under non-stressed conditions in the light–dark box assay. This is consistent with previous reports indicating that the anxiolytic efficacy of eCB augmentation is enhanced by anxiogenic or aversive environmental contexts^25,33,34^. MAGL inhibition, but not FAAH or dual FAAH/MAGL inhibition, was able to reduce novelty-induced anxiety-like behavior in the NIH assay, consistent with our previous report^30^. Although dual inhibition did not show any effects on anxiety-like behavior in light–dark box and NIH assays, it increased anxiety-like behaviors in OFT and these data are consistent with a previous report^35^. This suggests that dual FAAH/MAGL inhibition might increase anxiety-like behavior under basal conditions depending on experimental context.

Acute stress exposure decreases AEA and increases 2-AG levels in the brain^9,25,36^. This suggests that AEA signaling deficiency drives anxiety-like behavior and increased 2-AG represents a compensatory response aimed at counteracting stress-induced anxiety-like behaviors. Therefore, increasing eCB signaling could dampen stress-induced behavioral changes. From a therapeutic perspective, it is important to investigate whether eCB augmentation after stress exposure has occurred can reverse stress-induced anxiety-like behavior or if the eCB elevation must occur during the stress exposure. Our data indicated that either AEA or 2-AG, but not simultaneous AEA and 2-AG, augmentation before restraint exposure and after foot shock exposure attenuated restraint- and foot shock-induced decrease in the percent light time and light distance. Only 2-AG, but not AEA or simultaneous AEA and 2-AG, augmentation after foot shock stress exposure reduces the foot shock-induced increase in open arm immobility and latency to exit the open arm, and increases open arm entries and total distance traveled. Neither stress nor any treatment changed open arm time. Our data also demonstrated that simultaneous AEA and 2-AG augmentation could not reduce stress-induced anxiety-like behaviors when JZL195 was administered either before or after stress exposure. It is important to note that AEA augmentation was able to attenuate foot shock-induced anxiety in the light–dark box assay, consistent with our previous report ^27^, but not in the EZM. This might be due to the use of different mouse strains or could point to a complex paradigm specific drug effect. We used ICR mice in the light–dark box and C57BL/6j mice in the EZM experiment. This suggests that the effects of FAAH inhibition on anxiety-like behavior depend on the strain of mice, as well as testing environment.

In our previous report, we showed that acute stress increases spontaneous excitatory postsynaptic current (sEPSC) frequency onto BLA neurons, and sEPSC frequency was positively correlated with anxiety-like behavior. Both PF-3845 and JZL184 systemic administration decreases the stress-induced increase in sEPSC frequency^25^. This suggests that both PF-3845 and JZL184 act on CB1 receptors on the glutamatergic afferents to the amygdala to exert anxiolytic effects. However, a recent study by Di et al. reported that 2-AG signaling on GABAergic terminals within the amygdala contributes to anxiety-like behavior in the OFT, but not the elevated plus maze, after restraint stress exposure^37^. We found the opposite and have reproduced our previous results^25^ that 2-AG augmentation decreases anxiety-like behavior under non-stressed conditions in NIH, as well as after restraint and foot shock exposure in the light–dark and EZM, respectively. Moreover, our results are consistent with other studies indicating that 2-AG augmentation in the amygdala decreases anxiety-like behavior under non-stressed conditions^38^ and that 2-AG levels in the amygdala correlate with hypothalamic–pituitary–adrenal (HPA) axis habituation to repeated restraint stress^39^. Therefore, overall there is more support for the notion that 2-AG augmentation exerts anxiolytic effects.

The primary function of AEA and 2-AG signaling is the retrograde synaptic suppression of afferent neurotransmitter release within limbic brain structures including the amygdala^40–42^. The anxiolytic effects of low-dose cannabinoid agonist treatment are mediated through CB1Rs on forebrain glutamatergic, but not GABAergic terminals^15^. In contrast, the anxiogenic-like effects of high cannabinoid dose require activation of CB1Rs expressed on GABAergic neurons. Furthermore, deletion of CB1Rs from forebrain glutamatergic terminals produces increased fear behaviors^43^. Our data indicated that simultaneous augmentation of AEA and 2-AG increases anxiety-like behavior under non-stressed conditions. It has been shown that the anixogenic-like effects of JZL195 are CB1 mediated as the CB1 antagonist SR141716A blocked these effects^35^. This suggests that the anxiogenic-like effects of JZL195 might be due to a shift from CB1 activation of glutamatergic to GABAergic synapses. As CB1 receptors are more abundantly expressed on GABAergic than glutamatergic terminals, low doses of cannabinoids would be expected to activate CB1 receptor on GABAergic neurons first, thereby exerting anxiogenic-like effects. However, previous experiments clearly show that the anxiolytic-like effects of the low cannabinoid dose are mediated by the CB1 on glutamatergic terminals^15^. Therefore, it is possible that simultaneous augmentation of AEA and 2-AG mimics a high dose of cannabinoid treatment and results in activation of CB1Rs on GABAergic terminals in addition to glutamatergic terminals, which could provide a plausible explanation for the increased anxiety-like behavior produced by JZL195 treatment. Moreover, AEA is a partial CB1 agonist with higher receptor affinity and 2-AG is full CB1 agonist with lower receptor affinity^44^. Therefore, it is possible that AEA reduces the probability of 2-AG binding CB1 receptors on glutamatergic terminals, which are involved in the anxiolytic effects of selective 2-AG augmentation^25^. This could result in activating CB1 receptors on the GABAergic neurons. One more possibility is that AEA activates transient receptor potential vanilloid type-1 (TRPV1) receptors when pharmacologically increased 2-AG is in competition for CB1 binding. AEA can bind to TRPV1 receptors at higher concentrations^45^ and, in contrast with CB1 receptor activation, the activation of TRPV1 receptors has been shown to increase anxiety-like behaviors ^46^.

In accordance with previous reports, we found that the highest dose of JZL184 and both doses of JZL195 decrease the average velocity, vertical activity and number of fecal boli in the OFT^47^. Brain AEA and 2-AG levels in JZL195-treated mice and 2-AG levels in JZL184-treated mice were correlated with the total distance traveled, average velocity, number of fecal boli and vertical time in OFT. It seems likely that these effects in JZL195-treated mice are driven by 2-AG, rather than AEA, as similar effects were absent in the PF-3845, and present in JZL184, treated mice. We also noticed that JZL184 and JZL195 increased total distance traveled in the OFT, as well as in light–dark box assay. However, this is not surprising, as other studies have reported similar findings^25,48,49^. Initial studies reported that JZL184 suppresses locomotor activity^26,47^. However, the locomotor suppressant effects of JZL184 were not confirmed by subsequent studies; for example, JZL184 did not suppress locomotion in the elevated plus maze^34,50^, and JZL184 did not affect rotarod performance^51^. Overall, the effects of JZL195 and JZL184 on locomotor activity are intriguing and need further clarification. It should be noted that in general locomotor effects of cannabinoids are complex. Δ^9^-THC is well known to reduce locomotor activity. However, some studies have shown that it decreases locomotor activity in a sex-dependent manner, whereas others have shown that it has triphasic effects^52,53^. These findings are difficult to explain at present, but they suggest that the effects of MAGL and dual FAAH/MAGL inhibition are more complex than previously believed. These effects may depend on the testing environment, the time of testing, species/strains, etc.

Numerous data indicate that eCBs modulate cognitive processes in humans and in rodents^54^. There is general agreement that activation of the eCB system impairs learning and memory. A number of studies have demonstrated that direct (CB1 agonist Δ^9^-THC) and indirect (FAAH and MAGL inhibitor) activation of CB1 receptors can impair cognitive performance in a variety of memory assays^55–59^. The effects of memory impairments are more profound when FAAH and MAGL are inhibited simultaneously^60^. Although it has been shown that JZL184 impairs MWM performance in mice, it did so only at a dose (40 mg kg^–1^) that also inhibits FAAH ^50,60,61^.

However, the memory impairments by indirect activation of CB1 receptor (i.e., via FAAH and MAGL) inhibition often depend on dose. Conversely, some studies report memory-enhancing effects of eCB augmentation^54,60,62–64^. In accordance with other reports, we did not observe any memory impairment effects by PF-3845, JZL184 and JZL195 in MWM or Barnes-maze test at tested doses that affect anxiety-like behavior^60,64,65^. It should be noted that the drug administration did not occur during the training period and we examined the impact of AEA and/or 2-AG augmentation on memory recall and not on memory acquisition and consolidation. It remains possible that AEA and/or 2-AG augmentation could affect memory acquisition and consolidation. Although MWM is more stressful than Barnes-maze test, as it involves swimming for 6 consecutive days, we did not find major differences in the effects of PF-3845, JZL184 or JZL195 on the cognitive parameters. However, JZL184 decreased the target zone entries and JZL195 increased distance traveled in Barnes-maze test. This suggests that JZL184 and JZL195 produced a mild cognitive impairment in less stressful conditions but the same effects were not evident in the stressful MWM.

In conclusion, our studies suggest that MAGL inhibition could be an effective treatment not only as a preventative measure, but also after stress-related psychopathology has begun to manifest. However, FAAH inhibition could be an effective treatment as a preventative measure only, whereas dual inhibition of FAAH/MAGL is not likely to reduce stress-related affective dysfunction regardless of treatment timing. This suggests that dual FAAH/MAGL inhibition may not be a good strategy to treat mood and anxiety-related disorders.

## Electronic supplementary material

Supplementary material

Figure S1
