# Supplementary material for: Therapeutic endocannabinoid augmentation for mood and anxiety disorders: comparative profiling of FAAH, MAGL and dual inhibitors
Source: Transl Psychiatry. 2018 Apr 26;8:92. doi: 10.1038/s41398-018-0141-7 (PMC5917016; doi:10.1038/s41398-018-0141-7)
Supplement: Supplementary file 1 — Supplementary material [file 41398_2018_141_MOESM1_ESM.docx]

**Supplementary figure:**


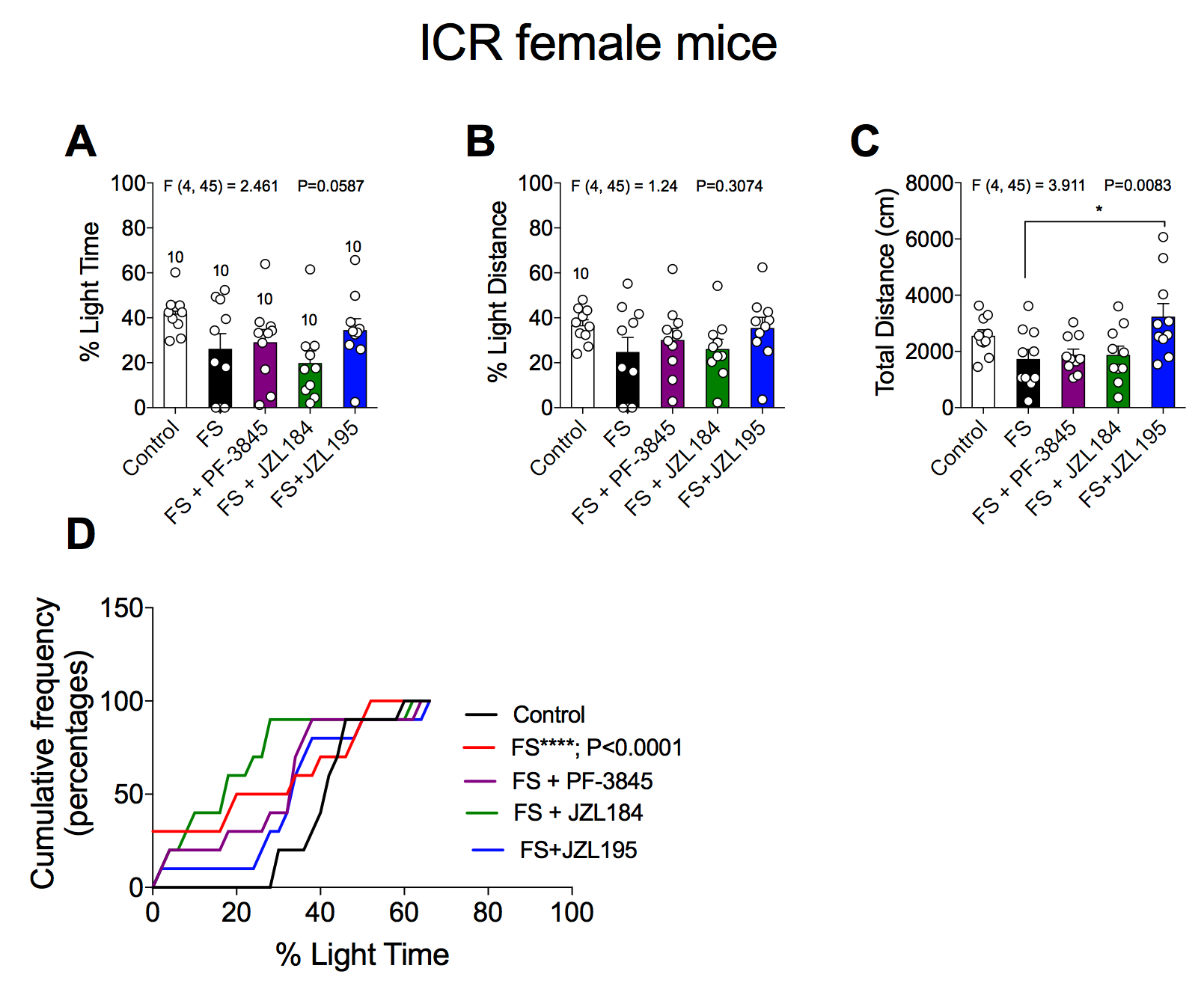


**Figure S1: Comparative effects of PF-3845, JZL184 and JZL195 on foot-shock induced anxiety-like behavior in the light-dark box in female ICR mice.**

The effects of PF-3845 (1 mg kg^-1^), JZL184 (10 mg kg^-1^) and JZL195 (10 mg kg^-1^) systemic administration on the (A) percent light time, (B) percent light distance and (C) total distance traveled in 10 minutes in the light-dark box assay. (D) Cumulative distribution curves depict the percentage of female mice at percent light time. F and p values from one-way analysis of variance noted above bar graphs. For cumulative frequency distributions, ****p<0.0001 *vs.* control group by Kolmogorov-Smirnov test. FS; foot shock.
