# Supplementary figures and images for: Therapeutic endocannabinoid augmentation for mood and anxiety disorders: comparative profiling of FAAH, MAGL and dual inhibitors
Source: Transl Psychiatry. 2018 Apr 26;8:92. doi: 10.1038/s41398-018-0141-7 (PMC5917016; doi:10.1038/s41398-018-0141-7)

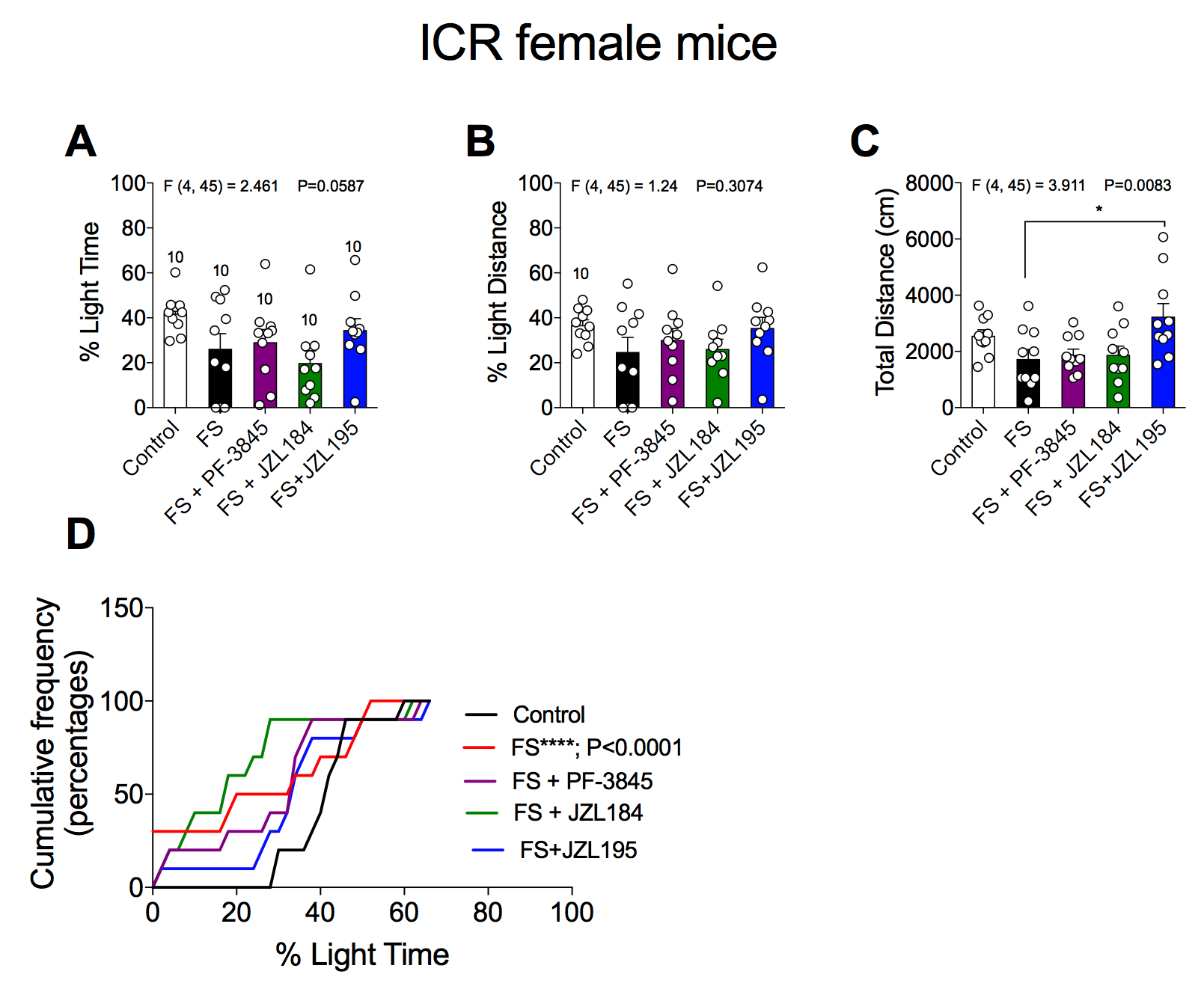

Supplement: Supplementary file 2 — Figure S1 [file 41398_2018_141_MOESM2_ESM.tif]
